# Development and study of the functional properties of marshmallow enriched with bee (*Apis mellifera*) honey and encapsulated probiotics (*Lactobacillus rhamnosus*)

**DOI:** 10.3389/fnut.2024.1353530

**Published:** 2024-04-18

**Authors:** Saira Itzel Colmenares-Cuevas, Adriana Contreras-Oliva, Josafhat Salinas-Ruiz, Juan Valente Hidalgo-Contreras, Enrique Flores-Andrade, Edgar Jesús García-Ramírez

**Affiliations:** ^1^Postgraduate College, Córdoba Campus, Amatlán de los Reyes, Mexico; ^2^Veracruzana University, Faculty of Chemical Sciences, Orizaba, Mexico

**Keywords:** antioxidants, bioactive compounds, coacervation, functional food, microencapsulation

## Abstract

Consumer demand for healthier confectionery products has prompted the confectionery industry to create products that are reduced in sugar content and supplemented with vitamins, antioxidants or biological elements beneficial to health. The aim of this study was to develop marshmallows enriched with *Apis mellifera* honey and *Lactobacillus rhamnosus* and to evaluate the effect of honey concentration and gelatin bloom degrees on marshmallow properties. A completely randomized design with a factorial structure was applied with different honey concentrations (0, 50 and 75%) and at different gelatin bloom degrees (265, 300 and 315 bloom degrees); moreover, the physicochemical properties, total phenol content and antioxidant activity of the marshmallow were studied, as well as the viability of the probiotic. The physicochemical properties of the marshmallows were found to be adequate and showed good stability over time. The concentration of honey and gelatin bloom degrees did not significantly affect probiotic viability. The density of the marshmallows decreased as the percentage of honey increased. Additionally, the pH was lower at higher honey concentrations. The marshmallow with 75% honey and 265 bloom degrees had a higher °Brix value. The honey treatments exhibited higher levels of total antioxidant activity and total phenolic compounds than the sugar-only marshmallows. However, the bloom degrees did not have a significant impact on the antioxidant activity and total phenolic compound content. Although the probiotics did not reach the minimum viability needed, their use as paraprobiotics can be considered.

## 1 Introduction

Confectionery products are classified as sugar confections and bakers’ confections; the former includes hard candies, soft candies and aerated candies (1). Among the aerated candies are marshmallows, which are masses of sugar, glucose or other sweeteners that are stabilized with protein substances such as albumins and gelatin (2).

The global confectionery market, valued in 2019 at US$201.3 billion, is growing at an annual rate of 3.6% and is projected to reach US$270.5 billion by 2027 (3); from a health point of view, this increase in the candy market is worrying, since the intake of added sugars is one of the main causes of obesity and diabetes in the world, so the rates of diseases related to sugar consumption may increase accordingly. However, the medicated confectionery segment offers functional products that in turn meet consumer demands for sweet products. In this regard, an annual growth rate of 4.1% is forecast for this segment during 2019–2027 (3).

Functional foods contain bioactive compounds that bring benefits beyond their nutritional properties. These contribute to improving physiological functions, preventing and/or mitigating the incidence of chronic diseases such as obesity, hypertension, diabetes and cancer (4). Within the classification of functional foods are foods or beverages to which a component beneficial to health has been added, such as omega-3, fiber, biological components, and antioxidants.

Among the beneficial components that can be added to functional foods is honey. Honey is a natural sweetener with various health benefits, including hepatoprotective, antioxidant, anti-obesity, hypolipidemic, and hypoglycemic properties. Honey also has anti-atherosclerotic, anti-cancer, hypotensive, immunomodulatory and neuroprotective effects (5). In addition, honey has a greater sweetening power than sugar, so it can partially or totally replace the use of sugar in confectionery products.

Other components that a functional food can include are probiotics, which are live microorganisms that, when administered in adequate amounts, generate a health benefit. Probiotics are often considered a safe and low-cost alternative for treating a variety of chronic diseases and improving overall human health. Probiotics are recognized for their ability to modulate host immunity and protect against several infectious and non-infectious pathologies. Some important probiotic attributes include pathogen killing, colonization, and host cell induction, which affect various host functions (6). To ensure that probiotics added to functional foods remain physiologically active at the time of consumption, it is necessary to maintain their stability and viability through technologies such as encapsulation techniques. These techniques protect probiotics from heat treatments, storage, and gastrointestinal conditions (7).

*Lactobacillus rhamnosus* GG (LGG) is a widely used probiotic strain with well-documented health effects. These include preventing and treating gastrointestinal infections and diarrhea, stimulating immune responses that promote vaccination, and potentially preventing certain allergic symptoms (8). Additionally, Szajewska and Hojsak (9) provided evidence that LGG can prevent antibiotic- or healthcare-associated diarrhea and reduce symptoms of acute gastroenteritis, mainly among children in Europe. Therefore, a marshmallow containing *L. rhamnosus* could be an attractive way for people of all ages to consume probiotics, particularly for children.

A few studies have been reported on the development of marshmallows with functional properties, all with the addition of extracts or compounds with antioxidant properties, but not with probiotics. Periche et al. (10) developed a marshmallow using stevia extracts, oligofructose, and isomaltulose as sugar replacers. They found that the sucrose and glucose syrup in commercial marshmallows could be replaced by a mixture of these replacers. Artamonova et al. (11) produced marshmallows using natural anthocyanin dyes derived from Sudanese rose and black chokeberry, resulting in high-quality characteristics. Santoso et al. (12) developed marshmallows with added kinang (chew of betel) extract, which exhibited antibacterial and antioxidant activity, as well as caries inhibition. Milea et al. (13) incorporated anthocyanins from sweet cherry skins in the development of marshmallows, resulting in increased anthocyanin content and antioxidant activity over time.

Based on the above, the aim of this work was to develop a marshmallow enriched with *Apis mellifera* bee honey and encapsulated *Lactobacillus rhamnosus*, as well as to evaluate its physicochemical properties, antioxidant capacity, phenolic compounds and probiotic viability.

## 2 Materials and methods

### 2.1 Materials used

We used the microorganism *Lactobacillus rhamnosus* obtained from a lyophilized strain (Vivolac, United States), broth culture medium and Man Rogosa Sharpe (MRS) agar (BD Difco, Mexico and Sigma Aldrich, United States), sodium alginate (Sigma Aldrich, USA), calcium chloride (CaCl_2_) (Química Mercurio, Mexico), multifloral honey from the *Apis mellifera* bee from the community of Zimatlán de Álvarez, gelatin at 265, 300 and 315 °Bloom (Diamante, Progel mexicana, S.A. de C. V), and standard sugar purchased at a supermarket.

### 2.2 Characterization of bee honey

Honey was harvested in accordance with AOAC 920.180 (2019). For the following analyses, the crystallized honey was placed in a water bath at a maximum temperature of 60°C until the crystals dissolved. These analyses determined color (14) with a photometer (HANNA, Model 96,785, Italy), pH with a potentiometer (Oakton Eco Testr pH 2, WD-35423-10, United States), total acidity (AOAC 962.19), ash (AOAC 920.181), electrical conductivity with a conductivity meter (HANNA, DiST3, Italy), moisture (AOAC 969.38 B) with a honey refractometer (Olimpo, RH-5890Be, China), reducing sugars (AOAC 920.183b), total soluble solids (AOAC 932.12) with a honey refractometer (Olimpo, RH-5890Be, China) and viscosity (Vibro Viscomer, model SV-10/SV-100, Japan).

#### 2.2.1 Hydroxymethylfurfural (HMF)

HMF is a yellow solid compound with great solubility in water, and its structure is a six-carbon heterocyclic structure with two functional groups: aldehyde and alcohol (hydroxymethyl). The amount of HMF present in honey is indicative of its freshness, and reflects its and storage length and conditions (15). The honey was heated in a water bath to 40°C to remove impurities and filtered. Five grams of sample was homogenized in 20 mL of distilled water, and HMF was measured at 25°C with a reflectometer (HANNA, RQflex 10, Italy).

#### 2.2.2 Extraction of bioactive compounds from honey

The amount of honey used for the extraction of bioactive compounds varied depending on the analysis to be performed. Two grams were used for the determination of antioxidant activity, and 1 g was used for the quantification of total phenolic compounds. Ten milliliters of acidified water (adjusted to pH 2 with 2 N HCl) was added to the sample (16), homogenized for 1 min using a vortex device and subjected to an ultrasonic bath (Auto science, Serial Ultrasonic Cleaner, 110 V/60 Hz, China) for 30 min at 25°C. Subsequently, the sample was left to macerate for 24 h in the dark at a temperature of 25°C. Afterward, the extract was centrifuged at 18.0 x g for 15 min at a temperature of 27°C, the supernatant was filtered, and the resulting product was preserved in amber tubes.

#### 2.2.3 Determination of antioxidant capacity (AC)

Antioxidant activity was determined using the 2,2-diphenyl-1-picrylhydrazyl (DPPH) synthetic free radical method (16). Absorbance was measured at a wavelength of 517 nm in a UV spectrophotometer (Thermo Fisher Scientific, Genesys 10-S, United States). A calibration curve was performed using Trolox at concentrations of 0–100 μg/mL in methanol. From the absorbance readings obtained, the antioxidant activity was calculated, and the results were expressed as mg Trolox/kg honey.

#### 2.2.4 Determination of total phenolic compounds (TPC)

The concentration of total phenolic compounds in honey was determined by the Folin–Ciocalteu method (16). Absorbance was measured in a UV spectrophotometer (Thermo Fisher Scientific, Genesys 10-S, United States) at a wavelength of 760 nm. A calibration curve was performed using gallic acid as a standard at concentrations of 0–100 mg/mL, and the results were expressed as mg gallic acid/kg honey.

### 2.3 Growth kinetics of *Lactobacillus rhamnosus*

The growth kinetics of Lactobacillus were obtained with the objective of determining the moment at which the largest number of viable cells was obtained to carry out the encapsulation of the microorganism. To determine the growth kinetics, 1% (v/v) *L. rhamnosus* inoculum was added to 100 mL of MRS broth previously sterilized (121°C for 15 min) and incubated (Riossa, ECF-82, Mexico) under anaerobic conditions at 32°C for 24 h, and samples were taken every 4 h. The pour plate method was applied using serial dilutions of each sample in triplicate. Colony count sensitivity ranged from 30 to 300 colonies. The Gompertz model equation was used to determine the kinetic parameters using SAS version 9.4 software.

### 2.4 Activation of *Lactobacillus rhamnosus*

Culture inoculum suspended in MRS broth with glycerol (1:1), which was stored at −20°C, was used. It was employed at a concentration of 1% (v/v) in MRS broth and incubated (Riossa, ECF-82. D, Mexico) under anaerobic conditions at a temperature of 32°C for 18 h (17). Subsequently, it was centrifuged (Eppendorf, Centrifuge 5,810 R, Germany) at 18 x g at 4°C for 20 min. The supernatant was filtered, and the cell concentrate was washed with sterile peptone water (0.1% w/v) several times to discard any residue from the culture medium (18).

### 2.5 Encapsulation of *Lactobacillus rhamnosus*

To prepare 100 g of encapsulation solution, 2% (w/w) sodium alginate, 0.2% gelatin, 5% nopal mucilage (1:2 w/v) and 92.8% distilled water were mixed (19). The solution was placed in an autoclave at 108°C for 5 min for sterilization. For encapsulation, work was carried out under sterile conditions; 9 mL of polymer mixture previously heated to 37°C was taken, and 1 g of lactobacillus cell concentrate was added. Then, the mixture was homogenized on a stirring plate at 400 rpm for 15 min and kept at rest for 1 h to achieve cross-linking of the capsules. Subsequently, the solution was filtered, and the collected capsules were washed with sterile distilled water, placed in a 0.1% sterile peptone water solution and stored at 4°C.

### 2.6 Making the marshmallows

The marshmallows were made following the methodology of the Federal Consumer Protection Agency (20) with some modifications. Table 1 shows the concentrations of sugar (100% = 47 g) and honey, as well as the bloom degrees of the gelatins used.

**Table 1:** Treatments applied for the preparation of marshmallows.

Treatment	Sugar concentration (%)	Honey concentration (%)	°Bloom
M1	100	0	265
M2	50	50	265
M3	25	75	265
M4	100	0	300
M5	50	50	300
M6	25	75	300
M7	100	0	315
M8	50	50	315
M9	25	75	315

To prepare the caramel, 5 g of glucose, sugar (depending on the treatment) and water in proportion to the amount of sugar (12 mL for 100% sugar) were heated to 121°C. On the other hand, to make the marshmallows, 5 mL of egg albumin was beaten at 280 rpm for 2 min using a mixer (KitchenAid, Artisan KSM150PSER, United States) until a shiny foam formed that adhered to the container. The speed was lowered to 180 rpm, and the previously prepared caramel was added. Once the caramel had been incorporated, the beating speed was increased to 280 rpm and maintained for another 2 min. Subsequently, gelatin (previously hydrated with water 1:5 w/v at 20°C for 10 min and heated to 80°C in a water bath) was added, beating at 225 rpm, and once incorporated, it was beaten at 280 rpm until the mixture cooled to a temperature of 40°C. Immediately afterward, honey preheated to 40°C was added, maintaining a beating speed of 280 rpm for 3 min; then, the beating speed was reduced to 180 rpm, and 10 g of probiotic capsules was added without stopping beating for another 2 min. The foam was then immediately poured into molds with corn starch, and a layer of starch was placed to cover the foam, which was left to dry for 24 h at 20°C. After this time, the excess cornstarch was removed from the marshmallows, and they were stored individually in polypropylene bags. The marshmallows were stored in a dry place at 25°C.

### 2.7 Physicochemical analyses of the marshmallows

Ash (AOAC 900.02 B), pH with a potentiometer (Oakton Eco Testr pH 2, WD-35423-10, United States), degrees Brix (AOAC 932.12) using a refractometer (Olimpo, RH-5890Be, China) and moisture (AOAC 925.45A) were determined.

#### 2.7.1 Density

The density of the marshmallows was determined in a cylindrical container with a known volume and weight. The foam was uniformly poured into the container and weighed to calculate its mass by weight difference and subsequently its density in g/mL.

#### 2.7.2 Extraction of bioactive compounds in marshmallow

For the determination of phenolic compounds and antioxidant capacity, extracts of the different marshmallow treatments were prepared. For this, freeze-dried samples were used at a vacuum pressure of 9.5 × 10 mm Hg for 24 h (10). Each extract was prepared by mixing 2 g of freeze-dried sample with acidified water (pH 2) at a 1:2 w/v ratio. This solution was homogenized in a vortex and subjected to ultrasonication for 30 min. It was then macerated in the dark for 24 h at a temperature of 25°C. After this time, the solution was centrifuged at 18.0 x g at 27°C for 20 min and finally filtered to remove the supernatant. The extracts were kept in amber tubes prior to use in the corresponding analyses.

#### 2.7.3 Determination of antioxidant capacity (AC)

To determine the antioxidant capacity in marshmallows, the same methodology used for honey was implemented (16), with modification of the resting conditions; in this case, they were in a water bath at 37°C for 30 min.

#### 2.7.4 Determination of total phenolic compounds (TPC)

The determination of total phenolic compounds was carried out using the Folin Ciocalteu method, according to the methodology of Cedeño-Pinos et al. (21), which was slightly modified. First, 0.25 mL of marshmallow extract was mixed with 7.75 mL of distilled water and 0.8 mL of 10% Folin–Ciocalteu reagent. This solution was left to stand for 8 min, and then 7.5% (w/v) Na_2_CO_3_ was added and homogenized. This mixture was left to stand in the dark at 25°C for 2 h. Finally, absorbance was determined at 760 nm in a spectrophotometer (Thermo Fisher Scientific, Genesys 10-S, USA).

#### 2.7.5 Viability of *Lactobacillus rhamnosus* encapsulated in marshmallow

To determine the viability of the microorganism, 1 g of marshmallow was added to 9 mL of 5% (w/v) sodium citrate, and then the mixture was stirred for 15 min to disintegrate the capsules and release the cells. The first dilution was performed with 1 mL of the solution in 9 mL of 0.1% peptone water. It was sown in Petri dishes by the pour plate technique from the 10^−5^ to 10^−8^ dilution in triplicate using MRS agar as culture medium. The dishes were incubated at 32°C for 48 h, and then cell counts were performed. The viability of the microorganisms was expressed as CFU/g marshmallow.

### 2.8 Statistical analysis

A completely randomized factorial design was applied for the preparation of the marshmallows, where Factor A was the honey concentration at 0, 50 and 75% and Factor B was the gel strength of the gelatin expressed in bloom degrees, which was 265, 300 and 315° bloom. To all treatments, 10% probiotic capsules (w/w) were added, corresponding to 3.17 × 10^9^ CFU/g marshmallow. To determine the influence of the factors on the physicochemical properties, total phenol content, antioxidant activity and viability of the probiotic (*L. rhamnosus*), ANOVA and a comparison of means with a 95% confidence level were performed using the GLIMMIX procedure of the SAS 9.4.3 statistical package.

## 3 Results and discussion

### 3.1 Physicochemical characteristics of bee honey

All the results obtained in the characterization of bee honey were within the values established in NOM-004-SAG/GAN-2018 (14). In relation to color, the honey presented a score of 9 on the Pfund scale, which corresponds to an “extra white” hue. Color in honey is defined by pigments such as phenolics, flavonoids, carotenoids, minerals and pollen. In this regard, it has been determined that darker honeys have a higher content of phenolic compounds (22).

On the other hand, the honey pH was 4.34, and its total acidity was 19.82 mEq acid/kg honey; when these quality parameters are outside standard values, they usually indicate sanitary deterioration (23). The ash content was 0.09%, which is explained by the fact that clear honeys have a low mineral content, while dark honeys contain higher mineral levels (24). The electrical conductivity of the honey was 0.23 mS/cm; this property is related to the soluble mineral content (25), since it is a technique for measuring the ability of a body or medium to conduct electric current.

On the other hand, honey moisture content was 17% and is related to the viscosity and crystallization properties, color, organoleptic properties and shelf life of honey (26). The reducing sugars in honey were 75.12%, while other honeys were in the range of 65–75% (27). The honey studied presented 81.5 °Brix, which is consistent with honeys from the state of Zacatecas, Mexico, ranging from 79.46 to 83.53 °Brix (28).

Additionally, the honey presented a viscosity of 26.1 Pa∙s; this parameter is affected by temperature, floral origin, water content and composition (29), while the HMF content was 5.57 mg/kg, which is an indicator of freshness since this compound is not present in fresh honey. Improper handling and storage conditions can also lead to its production (30); likewise, it increases when honey is heated to a high temperature (>100°C) in short periods of time (31).

Finally, the analyzed honey had an antioxidant capacity of 177.94 mg Trolox/kg. According to a study (32), the antioxidant content of honey produced by *Apis mellifera* in Central Serbia was 8.36 mg trolox/kg for acacia honey, 11.97 mg trolox/kg for polyfloral honey and 260.77 mg trolox/kg for forest honey. Furthermore, the total phenol content of the honey in the present study was 227.02 mg GA/kg; these results are somewhat low compared to other studies that have found phenol contents of 231 to 1,580 mg GA/100 g in polyfloral honeys (33), and 166.1 to 1019.2 mg GA/kg honey in honeys from the state of Hidalgo, Mexico (34).

### 3.2 Growth kinetics of *Lactobacillus rhamnosus*

**Figure 1:**
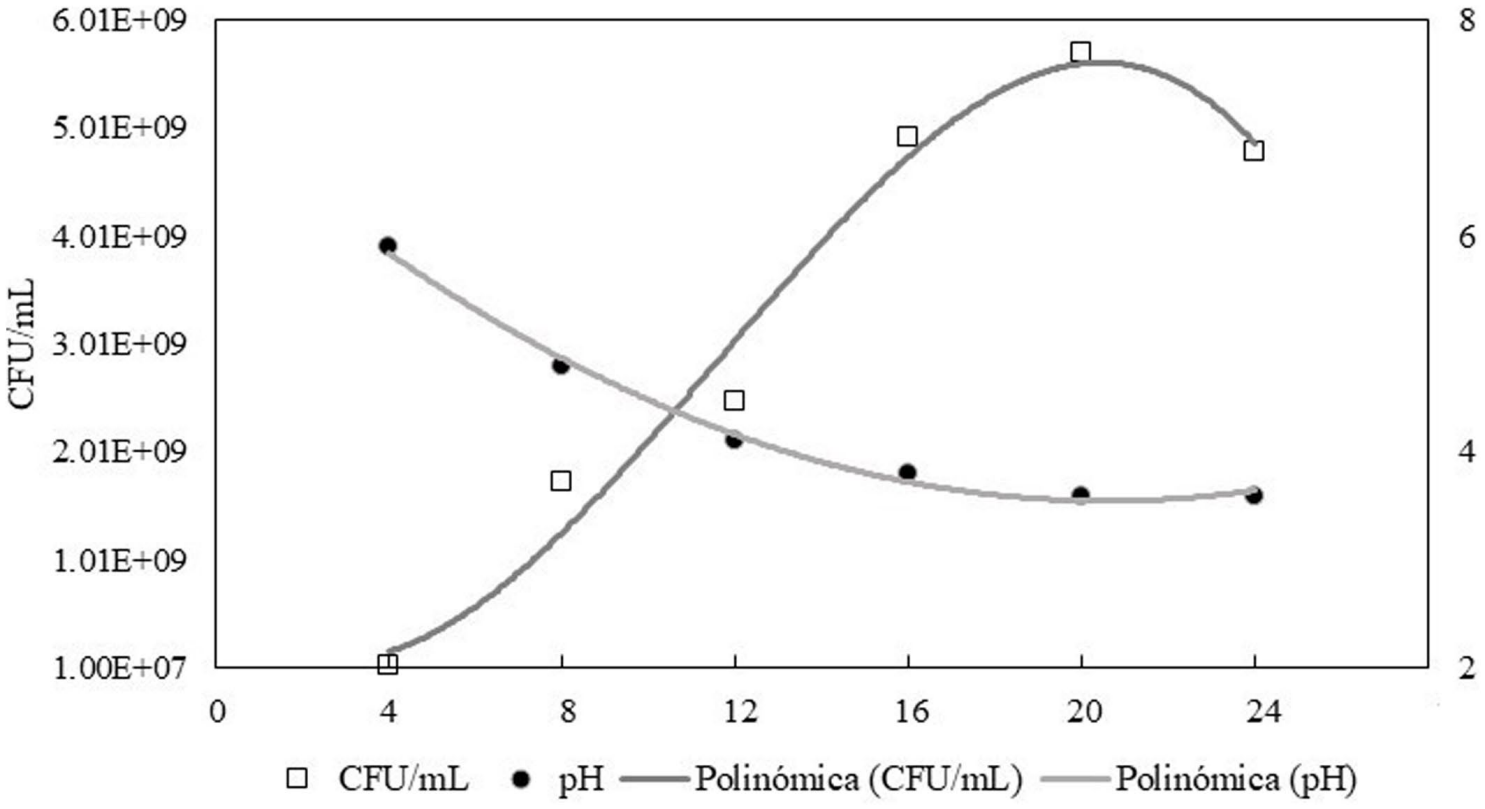
Growth kinetics of *L. rhamnosus* and pH variation in MRS culture medium at 32°C. CFU = Colony Forming Units.

Figure 1 shows the growth stages of the Lactobacillus *L. rhamnosus*. The beginning of the stationary phase is located at approximately 18 h of incubation, indicating the end of the exponential phase, so it was incubated at this time to prepare the inoculum to be encapsulated. In other studies, *Lactobacillus rhamnosus* B-445 inoculated at 37°C presented a higher bacterial population at 18 h (35), while at 22 h, there was a decrease in the rate of cell division (36), which is similar to what was observed in this study. On the other hand, the decrease in pH (6.1–3.6) is because *L. rhamnosus* is a lactic acid bacterium, so it can ferment carbohydrates present in the culture medium, resulting in the production of acids, mainly lactic acid (37).

### 3.3 Viability of encapsulated *Lactobacillus rhamnosus*

Oxygen has been found to be toxic to probiotics (38), so the encapsulation process may be useful to protect them, especially from the addition of air that is typical of the marshmallow making process. The *L. rhamnosus* inoculum was added to the encapsulating solution at a cell concentration of 3.5 × 10^10^ CFU/g beads, while the capsules finally showed a viability of 3.17 × 10^9^ CFU/g beads, so the encapsulation process did not affect the viability of the probiotic.

### 3.4 Marshmallow characterization

#### 3.4.1 Physicochemical characteristics

Table 2 shows the results of the physicochemical analyses of the different marshmallow treatments. In general, the marshmallows had ash contents of 0.43–0.57% and pH values of 3.50–5.05 and 60.00–65.50 °Brix. In particular, the honey concentration had no effect on the ash content of the marshmallows, so it is likely that the ash came from the probiotic capsules added to the marshmallows, which were made with alginate, gelatin and mucilage, in addition to having been subjected to a CaCl_2_ solution. The pH was lower at higher honey concentrations (*p* < 0.0001), with pH values ranging from 4.67 to 5.05 for marshmallows without honey, 3.75 to 3.8 for marshmallows with 50% honey, and 3.5 to 3.7 for marshmallows with 75% honey. These pH variations may be related to the content of organic acids present in the honey, so that the higher the honey concentration is, the higher the organic acid content. On the other hand, the bloom degrees of gelatin had an inversely proportional effect on the °Brix of the marshmallows, while the addition of honey increased the °Brix proportionally.

**Table 2:** Results of proximate analyses of marshmallow treatments expressed as means ± standard error.

	Concentration (%)		°Bloom	Density (g/mL)		Ash (%)		pH		°Brix	
	Honey	Sugar									
M1	0	100	265	0.43 ± 0.01	a	0.52 ± 0.002	bc	4.67 ± 0.07	b	60.33 ± 0.25	c
M2	50	50	265	0.42 ± 0.01	ab	0.57 ± 0.002	a	3.80 ± 0.08	c	61.00 ± 0.30	c
M3	75	25	265	0.40 ± 0.01	bc	0.56 ± 0.002	a	3.50 ± 0.08	d	65.50 ± 0.30	a
M4	0	100	300	0.44 ± 0.01	a	0.55 ± 0.002	ab	5.05 ± 0.08	a	60.00 ± 0.30	c
M5	50	50	300	0.38 ± 0.01	c	0.53 ± 0.002	abc	3.80 ± 0.08	c	60.00 ± 0.30	c
M6	75	25	300	0.35 ± 0.01	d	0.51 ± 0.002	bc	3.65 ± 0.08	cd	61.50 ± 0.30	b
M7	0	100	315	0.43 ± 0.01	a	0.49 ± 0.002	c	4.90 ± 0.08	ab	60.00 ± 0.30	c
M8	50	50	315	0.39 ± 0.01	c	0.44 ± 0.002	d	3.75 ± 0.08	cd	60.00 ± 0.30	c
M9	75	25	315	0.38 ± 0.01	c	0.43 ± 0.002	d	3.70 ± 0.12	c	60.00 ± 0.43	c

Means with the same letter indicate no significant difference based on Tukey’s test (α = 0.05).

The marshmallows had a density of 0.35–0.44 g/mL. The density decreased as the percentage of honey increased and that of sugar decreased and was higher in marshmallows with 265 °bloom gelatin. Sugar likely limits the amount of air entering the foam during the whipping process, so honey may be useful in providing desirable characteristics in marshmallows. In general, this property is important for foamy foods because it can define softness, lightness or porosity (39). Other studies have shown a similar density in marshmallows: 0.474 g/cm^3^ (40) and 0.41 g/mL (41).

**Figure 2:**
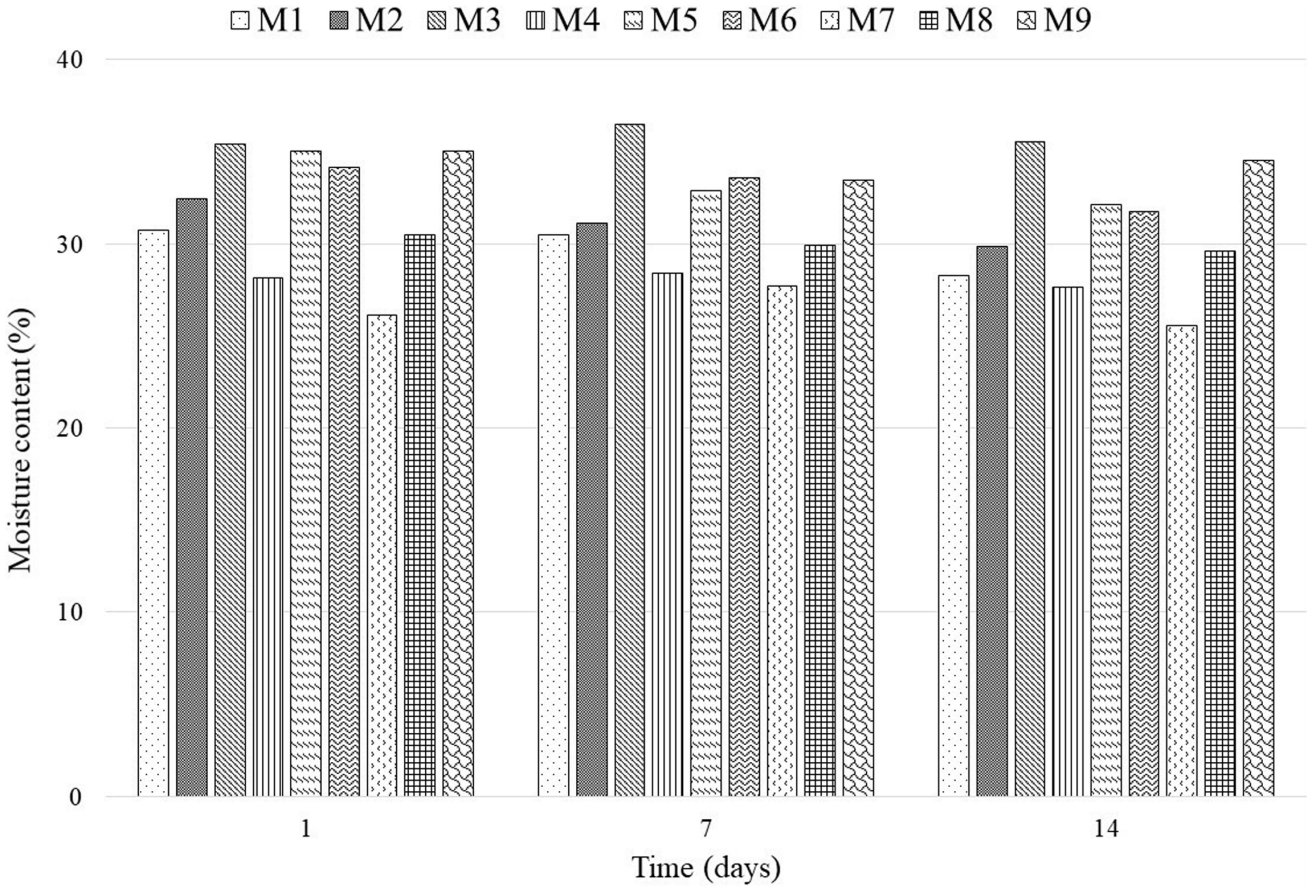
Moisture content of the different marshmallow treatments during storage time. M1, 0% honey-265 °bloom; M2, 50% honey-265 °bloom; M3, 75% honey-265 °bloom; M4, 0% honey-300 °bloom; M5, 50% honey-300 °bloom; M6, 75% honey-300 °bloom; M7, 0% honey-315 °bloom; M8, 50% honey-315 °bloom; M9, 75% honey-315 °bloom.

Figure 2 shows the moisture content of the marshmallow treatments on Days 1, 7 and 14 of storage. In general, moisture loss during storage was minimal and decreased with storage time in all samples, indicating the stability of the confection. Both physical and chemical aspects of freshness and stability during long-term storage can be affected by the moisture content. Even minor deviations from defined standards can have adverse effects on the physical properties of food (42). After 14 days of storage in a polypropylene wrapper, the moisture content of the marshmallows without honey was 25 to 35%, and those with the highest amount of honey (75%) had 32 to 36% moisture. Other authors have reported different moisture values in confectionery products with functional properties: 19 to 21.5% in marshmallows with anthocyanins (11); 16.7 to 22.9% in marshmallows with *Stevia rebaudiana*, oligofructose and isomaltose as sugar substitutes (10); 22.06 to 22.55% in jelly candies with rosemary extract (21); and 20.05 to 25.3% in jelly candies with honey and fruit juices (43). The marshmallows with probiotics had a higher moisture content than the aforementioned confections, probably due to the content of the probiotic beads.

#### 3.4.2 Antioxidant capacity (AC)

**Figure 3:**
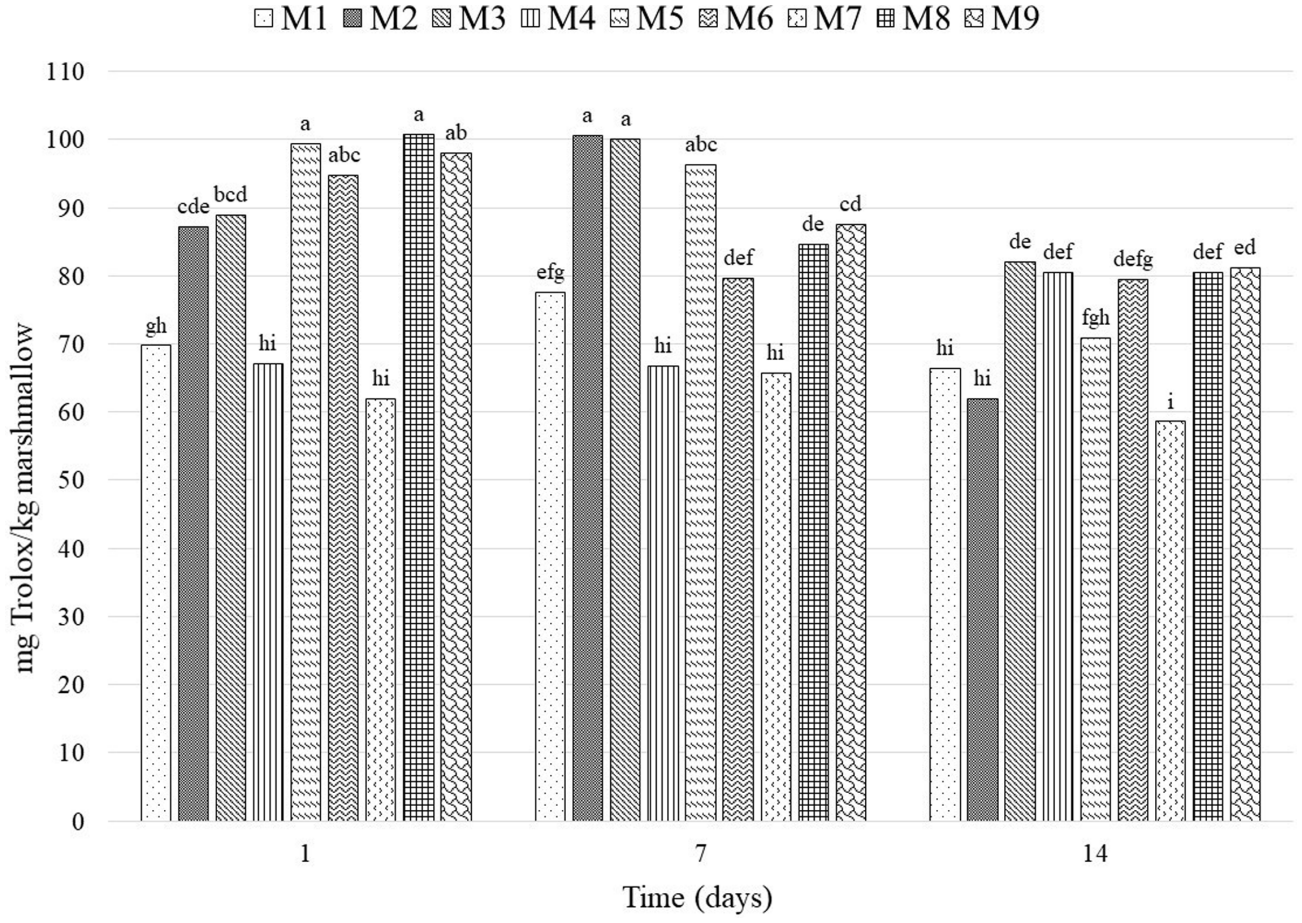
Antioxidant activity of marshmallow treatments on different days of storage. Means with the same letter do not present a significant statistical difference (α = 0.05). M1, 0% honey-265 °bloom; M2, 50% honey-265 °bloom; M3, 75% honey-265 °bloom; M4, 0% honey-300 °bloom; M5, 50% honey-300 °bloom; M6, 75% honey-300 °bloom; M7, 0% honey-315 °bloom; M8, 50% honey-315 °bloom; M9, 75% honey-315 °bloom.

Figure 3 shows the AC of the marshmallow treatments during storage. Antioxidant activity was not affected by the gelatin bloom degree; however, honey concentration had a significant effect on the treatments (*p* < 0.0001), as well as the interaction between the two factors (p < 0.0001). The marshmallows presented AC values from 61.87 to 100.81 mg Trolox/kg marshmallow. In general, it can be observed that marshmallows with honey presented more AC than those without honey, being less noticeable on Day 14, which may be due to a slight survival of probiotics, since strains of lactobacilli and some bididobacteria possess AC and are able to decrease the risk of free radical accumulation (44). Furthermore, Sharma et al. (45) found that *L. rhamnosus* GG had an inhibition percentage of 59%. Therefore, AC in marshmallows is likely influenced by probiotic content (21).

At the end of the experiment (Day 14), treatment M7 (0% honey-315 bloom) presented the lowest AC with 58.58 mg Trolox/kg, while M3 (75% honey-265 bloom) had the highest AC with 82.02 mg Trolox/kg marshmallow. Periche et al. (10) made marshmallows with an aqueous extract of Stevia that had an AC of 117 mg Trolox/100 g aqueous extract, while Santoso et al. (12) prepared marshmallows with *Kinang* extract, and the treatment with 80% extract presented 2.78 mg/mL antioxidant activity.

#### 3.4.3 Total phenolic compounds (TPC)

**Figure 4:**
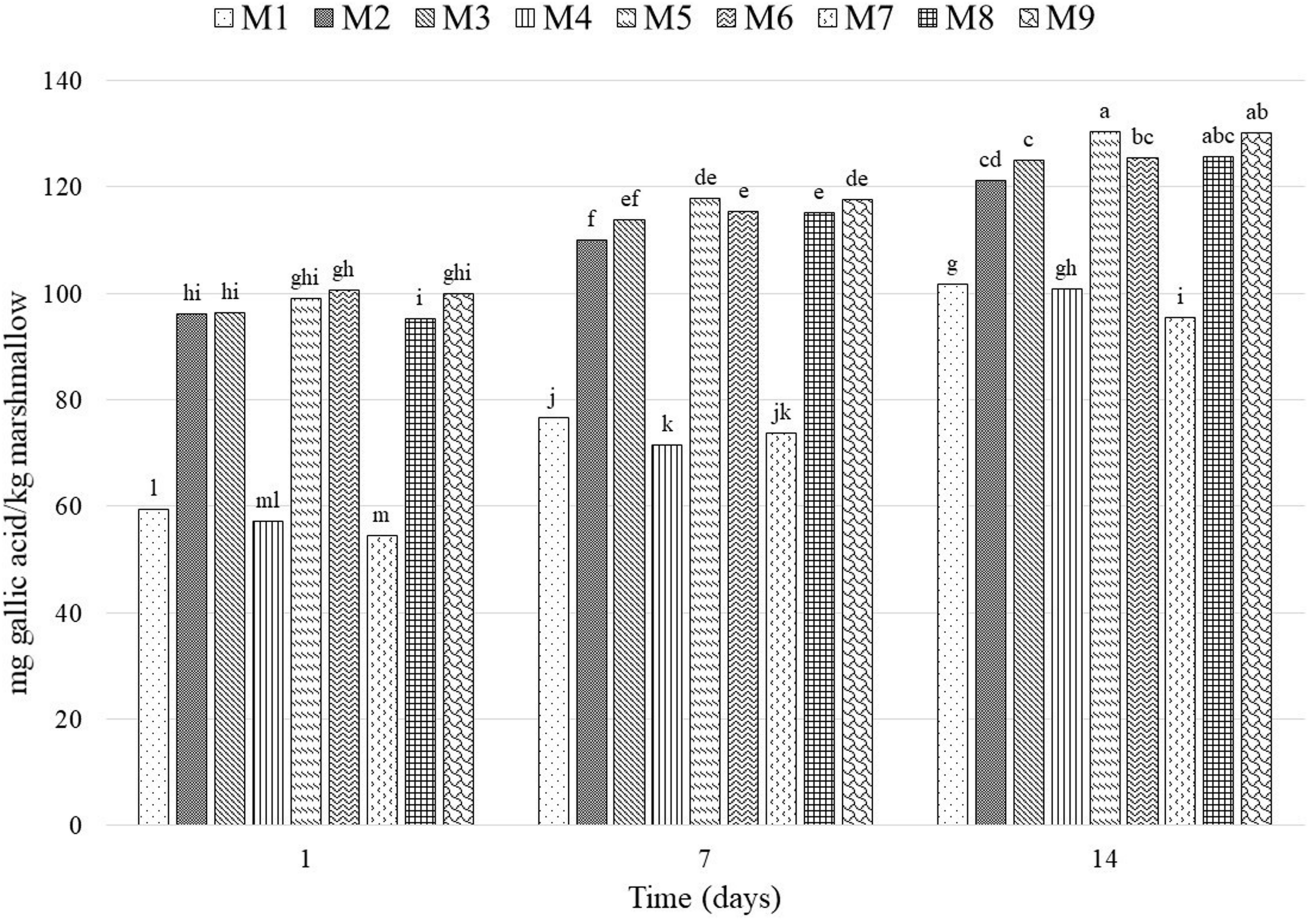
Total phenol content of marshmallow treatments. Means with the same letter do not show a significant statistical difference (α = 0.05). M1, 0% honey-265 °bloom; M2, 50% honey-265 °bloom; M3, 75% honey-265 °bloom; M4, 0% honey-300 °bloom; M5, 50% honey-300 °bloom; M6, 75% honey-300 °bloom; M7, 0% honey-315 °bloom; M8, 50% honey-315 °bloom; M9, 75% honey-315 °bloom.

The honey concentration in the marshmallows presented a significant effect (*p* < 0.0001) on the total phenol content. Although bloom degrees did not present a significant effect, the interaction of the two factors had a significant influence (*p* < 0.0001). Figure 4 shows that the honey treatments had a higher phenol content, which is explained by the TPC of the honey added to the marshmallows (386 GA/kg). In general, the concentration of phenolic compounds increased with storage time.

On Day 1, treatment M7 (0% honey-315°bloom) showed the lowest phenol content, with 54.45 mg GA/kg marshmallow, while M6 (75% honey-300°bloom) had a content of 100.55 mg GA/kg marshmallow. At the end of the experiment (Day 14), M7 (0% honey-315°bloom) again showed the lowest phenol content, with 95.33 mg GA/kg marshmallow, and the treatment with the highest concentration was M5 (50% honey-300°bloom) with 130.4 mg GA/kg marshmallow. Cedeño-Pinos et al. (21) analyzed jelly candies enriched with a rosemary extract with 73.9 mg polyphenols/g; however, the candies had polyphenol contents from 0.19 to 0.41 mg/g; this indicates that the phenol content can be easily lost over storage time, so it is important that the phenol source has a significant amount for the best results.

#### 3.4.4 Viability of *Lactobacillus rhamnosus*

**Figure 5:**
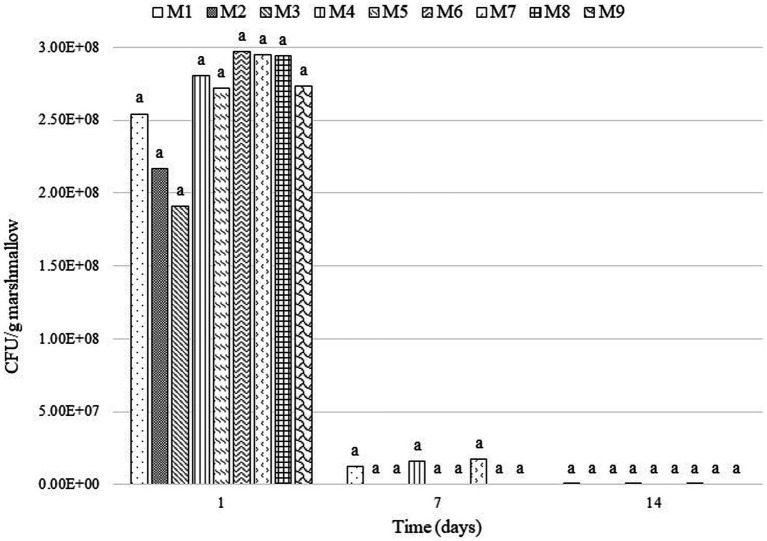
Viability of *L. rhamnosus* in marshmallow formulations during the storage period. Means with the same letter do not show a statistically significant difference (α = 0.05). CFU=Colony Forming Units. M1, 0% honey-265 °bloom; M2, 50% honey-265 °bloom; M3, 75% honey-265 °bloom; M4, 0% honey-300 °bloom; M5, 50% honey-300 °bloom; M6, 75% honey-300 °bloom; M7, 0% honey-315 °bloom; M8, 50% honey-315 °bloom; M9, 75% honey-315 °bloom.

Figure 5 presents the results of the viability analysis of the probiotic *L. rhamnosus* incorporated in the marshmallows. On the first day of storage, the viability of the lactobacilli remained above the minimum recommended limit (10^6^ CFU/g) (46) in all formulations. However, by Day 7, viability was reduced from 4.8 to 6.0% in marshmallows without honey, which presented 1.23 × 10^7^, 1.58 × 10^7^ and 1.76 × 10^7^ CFU/g marshmallow for treatments with 265, 300 and 315°bloom, respectively, while marshmallows with 50 and 75% honey had a drastic reduction in CFU/g marshmallow on this same day. Finally, by Day 14, none of the treatments reached minimum viability.

Lactobacillus survival can be influenced by several factors, such as storage temperature, water activity (a_w_), titratable acidity, presence of sugars, and processing conditions (heat treatment, cooling rate, packaging material, etc.) (47). On the other hand, pH limits the growth and stability of probiotic bacteria (47). The acidification of the medium due to lactic-acid bacteria growth poses a challenge for industrial production. The accumulation of lactic acid may affect cell physiology, leading to growth interruption or reduction. Therefore, maintaining a pH close to neutrality can promote a higher growth rate (48); however, the pH of the marshmallows varied from 3.50 to 5.05, being lower in the treatments with honey, since it showed a pH of 4.34, so that on Day 7, the viability of the marshmallows with honey was lower than that of the treatments without it.

On the other hand, another important factor in the viability of probiotics is acidity. This characteristic is due to the presence of organic acids, among which gluconic acid (~0.5% w/v) is important in honey; it is generated from the oxidation of glucose by the endogenous glucose oxidase enzyme and is a very powerful antibacterial agent. In addition, when honey is diluted, glucose oxidase is activated and acts on endogenous glucose to produce hydrogen peroxide (H_2_O_2_), which has antibacterial properties (49, 50). The antimicrobial activity of honey derived from low water activity (0.56 to 0.62) and its high viscosity, among other characteristics, can also be considered (51).

High concentrations of sugars in marshmallows could trigger the osmosis process in probiotics, causing water loss from bacterial cells and affecting bacterial growth (52). Additionally, the survival of probiotics can be affected by the characteristics of the packaging material, such as thickness and gas permeability (53).

According to the viability results of the marshmallows, the applied encapsulation was not able to protect the microorganisms from the mentioned factors. However, these probiotics fall within the classification of paraprobiotics, also called inactivated probiotics or ghost probiotics, which are inactivated cells or fractions of microbial cells that provide a benefit to the host at appropriate doses (54). Therefore, they could be used in more vulnerable patients without risk (55).

Shigwedha et al. (56) showed that the administration of inactivated *L. rhamnosus* V. probiotics is useful in the prevention and treatment of infectious diseases, allergies, fatigue and fibromyalgia, whereas Good et al. (57) demonstrated that oral administration of UV-inactivated *L. rhamnosus* HN001 attenuates the severity of necrotizing enterocolitis in neonatal mice and preterm piglets. Although the project aimed to preserve the viability of probiotics in marshmallows, more time is required to test other encapsulation methods and materials, so there are opportunities for future research. However, based on the above, it is possible that the marshmallows produced in this study could be studied by researchers in the area of health as a supplement with potential for the prevention of cancer and other gastrointestinal system conditions. On the other hand, the project made it possible to obtain marshmallows with antioxidant activity and phenolic compounds derived from *Apis mellifera* honey, thus obtaining a product with functional properties.

## 4 Conclusion

Marshmallows enriched with honey and *L. rhamnosus* probiotics were prepared, and their physicochemical properties, antioxidants and probiotic viability were studied; their physicochemical properties were adequate and showed good stability over time. The antioxidant capacity and total phenol content of the marshmallows were increased by the addition of honey, creating a functional food; furthermore, the bloom degrees did not affect the antioxidant capacity or the amount of total phenols in the marshmallows. Although the antioxidant activity and phenolic compounds were preserved during the storage time, the viability of the probiotics was not maintained despite being encapsulated; however, it is possible that the dead probiotics may still exert a beneficial effect on the consumer by acting as paraprobiotics.

## Data availability statement

The raw data supporting the conclusions of this article will be made available by the authors, without undue reservation.

## Author contributions

SC-C: Writing – original draft, Visualization, Investigation. AC-O: Writing – review & editing, Validation, Supervision, Resources, Project administration, Methodology, Conceptualization. JS-R: Writing – review & editing, Formal analysis. JH-C: Writing – review & editing, Formal analysis. EF-A: Writing – review & editing, Supervision. EG-R: Writing – review & editing, Methodology, Investigation.
